# Inhibition of highly pathogenic avian influenza (HPAI) virus by a peptide derived from vFLIP through its direct destabilization of viruses

**DOI:** 10.1038/s41598-017-04777-4

**Published:** 2017-07-07

**Authors:** Ho-Jin Moon, Chamilani Nikapitiya, Hyun-Cheol Lee, Min-Eun Park, Jae-Hoon Kim, Tae-Hwan Kim, Ji-Eun Yoon, Won-Kyung Cho, Jin Yeul Ma, Chul-Joong Kim, Jae U. Jung, Jong-Soo Lee

**Affiliations:** 1 College of Veterinary Medicine, Chungnam National University, Daejeon, Republic of Korea; 2 Department of Biomedical Sciences, College of Veterinary Medicine, Iowa State University, Ames, IA USA; 3 Animal and Plant Quarantine Agency, 175 Anyang-ro, Manangu, Anyang City, Gyeonggido 430-757 Republic of Korea; 4 Korean Medicine (KM) Application Centre, Korea Institute of Oriental Medicine, Daegu, 41062 Republic of Korea; 5 Department of Molecular Microbiology and Immunology, University of Southern California, Keck School of Medicine, Los Angeles, CA USA

## Abstract

The antiviral activities of synthesized Kα2-helix peptide, which was derived from the viral FLICE-like inhibitor protein (vFLIP) of Kaposi’s sarcoma-associated herpesvirus (KSHV), against influenza A virus (IAV) were investigated *in vitro* and *in vivo*, and mechanisms of action were suggested. In addition to the robust autophagy activity of the Kα2-helix peptide, the present study showed that treatment with the Kα2 peptide fused with the TAT peptide significantly inhibited IAV replication and transmission. Moreover, TAT-Kα2 peptide protected the mice, that were challenged with lethal doses of highly pathogenic influenza A H5N1 or H1N1 viruses. Mechanistically, we found that TAT-Kα2 peptide destabilized the viral membranes, depending on their lipid composition of the viral envelop. In addition to IAV, the Kα2 peptide inhibited infections with enveloped viruses, such as Vesicular Stomatitis Virus (VSV) and Respiratory Syncytial Virus (RSV), without cytotoxicity. These results suggest that TAT-Kα2 peptide is a potential antiviral agent for controlling emerging or re-emerging enveloped viruses, particularly diverse subtypes of IAVs.

## Introduction

Seasonal influenza A virus (IAV), a RNA virus of the Orthomyxoviridae family, presents a considerable threat to human health in terms of both morbidity (with 2–5 million cases of severe illness per year) and mortality (with 250,000–500,000 deaths per year) worldwide^1, 2^. Although vaccination is one of the proven method for the control of infectious diseases, antigenic drift among influenza viruses means that vaccines need to be reformulated in every year to provide strain specific immunity, and this reformulation process is complex, costly and time consuming. Thus, the researchers have sought to produce universal influenza virus vaccines to provide extended or even lifelong protection against broad spectrum of influenza virus^3–10^. In addition to these efforts, development of new antiviral agents with high efficacy is crucial for situations where no effective flu vaccine is available for use against pandemic strains, which arise at irregular intervals. For example, antiviral drugs such as oseltamivir and zanamivir were reported to be effective against the H1N1 pandemic in 2009–2010^11^. However, increases in the number of influenza strains that are resistant to currently available antiviral drugs, as well as the occurrence of adverse effects with some available drugs^12^, necessitates the development of new stratergies for controlling virus infections. Furthermore, novel prophylactic options are still necessary for immunocompromised individuals, such as very young and older populations^13^. Therefore, the continuous research are required for the investigation of effective preventive and controlling methods against these viruses.

Natural antimicrobial peptides (AMP) are reported to have diverse antimicrobial activities against bacteria, fungi, viruses and parasites^14^. Natural peptides or synthetic counterparts represent a new generation of antiviral agents with properties of selectivity and specifity, low level of adverse effects and low probability of developing resistance during the treatment^15^. Common features of the antimicrobial peptides are small size, positive charge of 2–9 and an amphipathic structure^16^. These peptides have been shown to have various therap﻿euti﻿c properties against infection by a number of pathogens, including viruses^17^. The mechanisms of action depend on the peptide structures, and activities can be improved by modification of the native peptides or their synthetic counterparts.

Previously Lee *et al*., reported that Kα2, derived from viral FLICE-like inhibitor protein (vFLIP) of Kaposi’s sarcoma-associated herpesvirus (KSHV) as a robust autophagy inducer, that inhibits the binding of FLIP to the E2-like enzyme Atg3 without affecting the interaction with microtubule-associated protein 1A/1B-light chain (LC3) and Atg3^18^. The synthetic Kα2 peptide, which consists of 10 amino acids [EVVLFLLNVF] from the α2 helix of death effector domain 1 (DED1) of KSHV (also known as human herpesvirus 8), was fused with the protein transduction domain [YGRKKRRQRRR] of the TAT protein of HIV-1 to localise the TAT-Kα2 fusion peptide toward the inner side of the liposome of influenza virus through the lipid bilayer^18^.

TAT-Kα2 is a small, cationic, hydrophobic peptide, and we hypothesised that it might possess antipathogenic activity. We therefore investigated the antiviral activity of TAT-Kα2 against highly pathogenic avian influenza (HPAI) strains and other enveloped RNA and DNA viruses *in vitro*, and found that this activity results from virucidal effects of the peptide. Additionally, we demonstrated the antiviral activity of TAT-Kα2 against pathogenic influenza virus strains H1N1 and H5N1 *in vivo* in a mouse model, and explain its mode of action is also due to the virucidal activity.

## Results

### The TAT-Kα2 peptide is not cytotoxic

**Figure 1:**
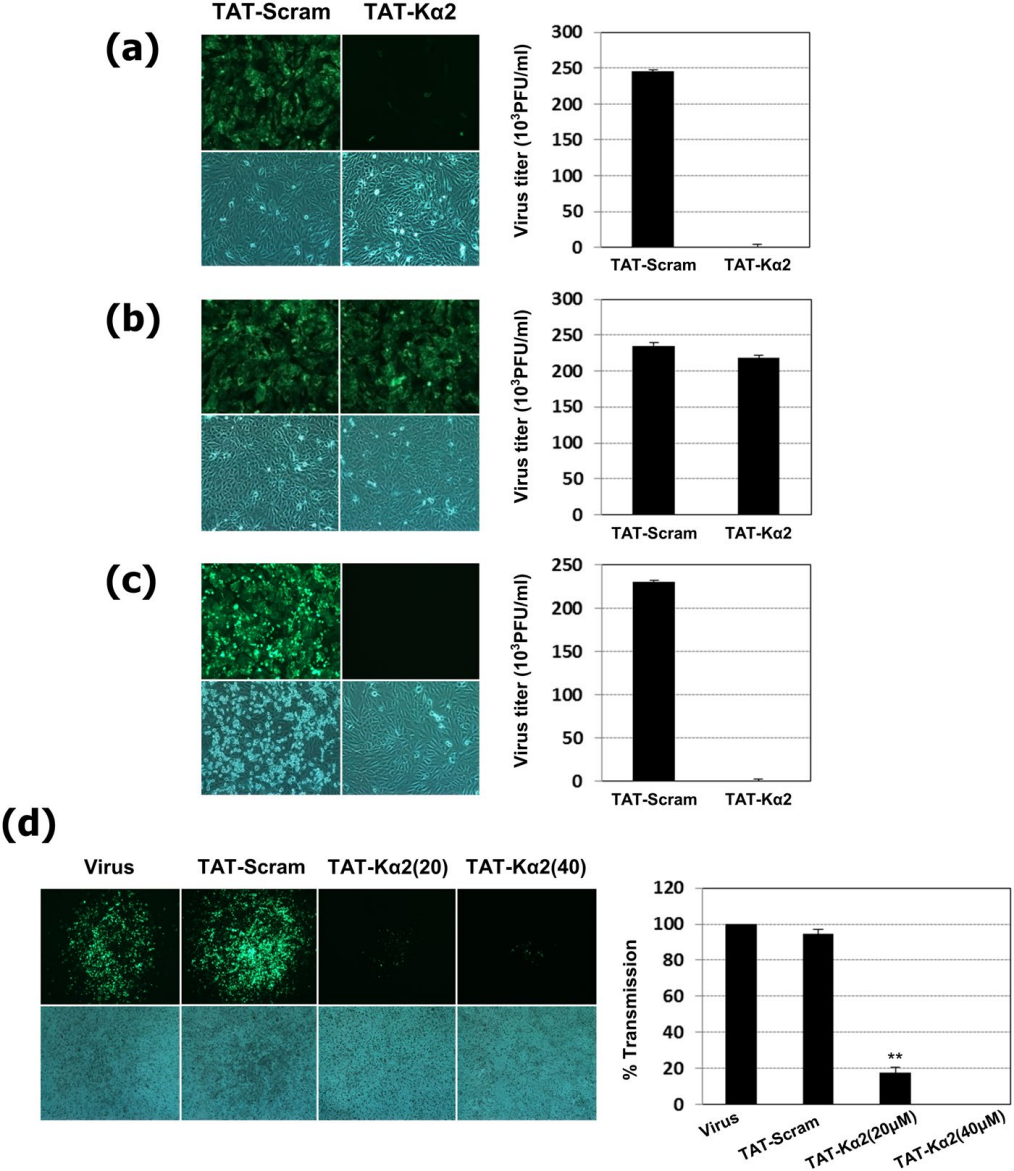
Anti-influenza effect of the TAT-Kα2 peptide and determination of the critical stage of virus infection for its antiviral action. Three different time points of treatment with the TAT-Kα2 and TAT-scramble peptides were compared to determine which step of virus infection is critical for inhibition. (**a**) Attachment step. The peptide and PR8 virus expressing GFP (PR8-GFP) were simultaneously added to MDCK cells. (**b**) Entry step. The cells were infected with the PR8-GFP virus for 1 h prior to the peptide treatment. (**c**) Pre-attachment step. Each peptide was pre-incubated with the PR8-GFP virus for 20 min at RT. For (**a**–**c**), the infected cells were detected two days later using GFP. The plaques were counted, and the virus titers are shown in the graph. (**d**) Transmission inhibition assay. The cells that were treated with virus only, the TAT-scramble peptide, and two different concentrations of the TAT-Kα2 peptide (20 and 40 μM) were compared to determine their capacity to inhibit virus transmission. The results are shown in the graph as percent transmission and were normalized to the 100% transmission of the virus only group.

The previous study by Lee *et al*.^18^ shed light on the potential of the TAT-Kα2 peptide to elicit innate immunity against invasive pathogens, such as viruses. Here, for the purpose of a preliminary evaluation of its antiviral efficacy, different concentrations of the TAT-Kα2 peptide were tested in Madin-Darby canine kidney (MDCK) cells to determine whether the TAT-Kα2 peptide was cytotoxic. No cytotoxicity was observed, even for concentrations upto 40 µM (Fig. 1d) and no cytopathic effect (CPE) was observed.

### The TAT-Kα2 peptide has anti-influenza virus activity at the initial stages of influenza virus infection

When MDCK cells were infected with a TAT-Kα2-treated influenza virus (A/Puerto Rico/8/34, PR8) expressing green fluorescent protein (PR8-GFP), infection of the MDCK cells was strongly diminished relative to infection with untreated virus or virus treated with the TAT peptide fused to a scrambled-sequence peptide (TAT-scramble; Fig. S1A; see Table 1 for the peptide sequence). Consequently, the antiviral action of TAT-Kα2 at critical steps in influenza virus infection, such as pre-attachment (binding between hemaglutinnin [HA] and cellular sialic acid), attachment, and post-attachment (endocytosis) was determined. For the pre-attachment assay, PR8-GFP virus (multiplicity of infection [MOI] of 0.5) and TAT-Kα2 peptide (12 μM) were pre-incubated for 20 min at room temperature (RT) prior to cell treatment. For the attachment step, PR8-GFP virus and TAT-Kα2 peptide were simultaneously added to the cells. For the post-attachment assay, the virus was incubated with the cells for 1 h at 4 °C and washed prior to the TAT-Kα2 peptide treatment. Notably, when the virus was pre-treated with the TAT-Kα2 peptide, the results clearly showed the antiviral activity of the peptide during the pre-attachment step (Fig. 1c). Moreover, as shown in Fig. 1a, the simultaneous treatment also resulted in the inhibition of virus infection, whereas the TAT-scramble peptide (the amino acid sequences are indicated in Table 1) did not inhibit infection. However, during the post-virus attachment step, the TAT-Kα2 peptide was unable to inhibit virus infection and subsequent replication (Fig. 1b). These findings suggest that the TAT-Kα2 peptide blocks the pre-attachment step or the moment of attachment in the PR8 influenza virus life cycle, suggesting that this peptide has antiviral properties.

**Table 1:** Synthesized peptides in this study.

Peptides	Sequence (Retro-Inverso Peptide [D-isomer])
TAT	R R R Q R R K K R G Y
TAT-Scramble	R R R Q R R K K R G Y G K K N E K R K K D K V K N R
TAT-Kα2	R R R Q R R K K R G Y G F V N L L F L V V E
TAT-Kα2-M	R R R Q R R K K R G Y G F V N L **A A A** V V E
TAT-Kα2-M1	R R R Q R R K K R G Y G F V N L **A** F L V V E
TAT-Kα2-M2	R R R Q R R K K R G Y G F V N L L **A** L V V E
TAT-Kα2-M3	R R R Q R R K K R G Y G F V N L L F **A** V V E

### Transmission of the influenza virus was inhibited by the TAT-Kα2 peptide *in vitro*

To further understand the capacity of the peptide to inhibit viral transmission, an *in vitro* assessment was performed. Cells were infected with PR8-GFP virus (0.5 MOI) for 2 h to enable the virus to attach completely, and an overlay media containing the TAT-Kα2 peptide was then added. With overlay medium containing no peptide or containing TAT-scramble, virus transmission (repeated infection to neighbouring cells after viral budding) was observed, however, with overlay medium containing 20 μM or 40 μM TAT-Kα2, virus transmission was suppressed (Fig. 1d). These results indicate that TAT-Kα2 peptide can critically inhibit the initial steps of the IAV life cycle, particularly the steps occurring prior to or at virus-cell attachment, thereby suppressing the interaction between the cell and virus, and inhibiting the spread of infectious viruses.

### TAT-Kα2 destabilises the structure of IAV particles

**Figure 2:**
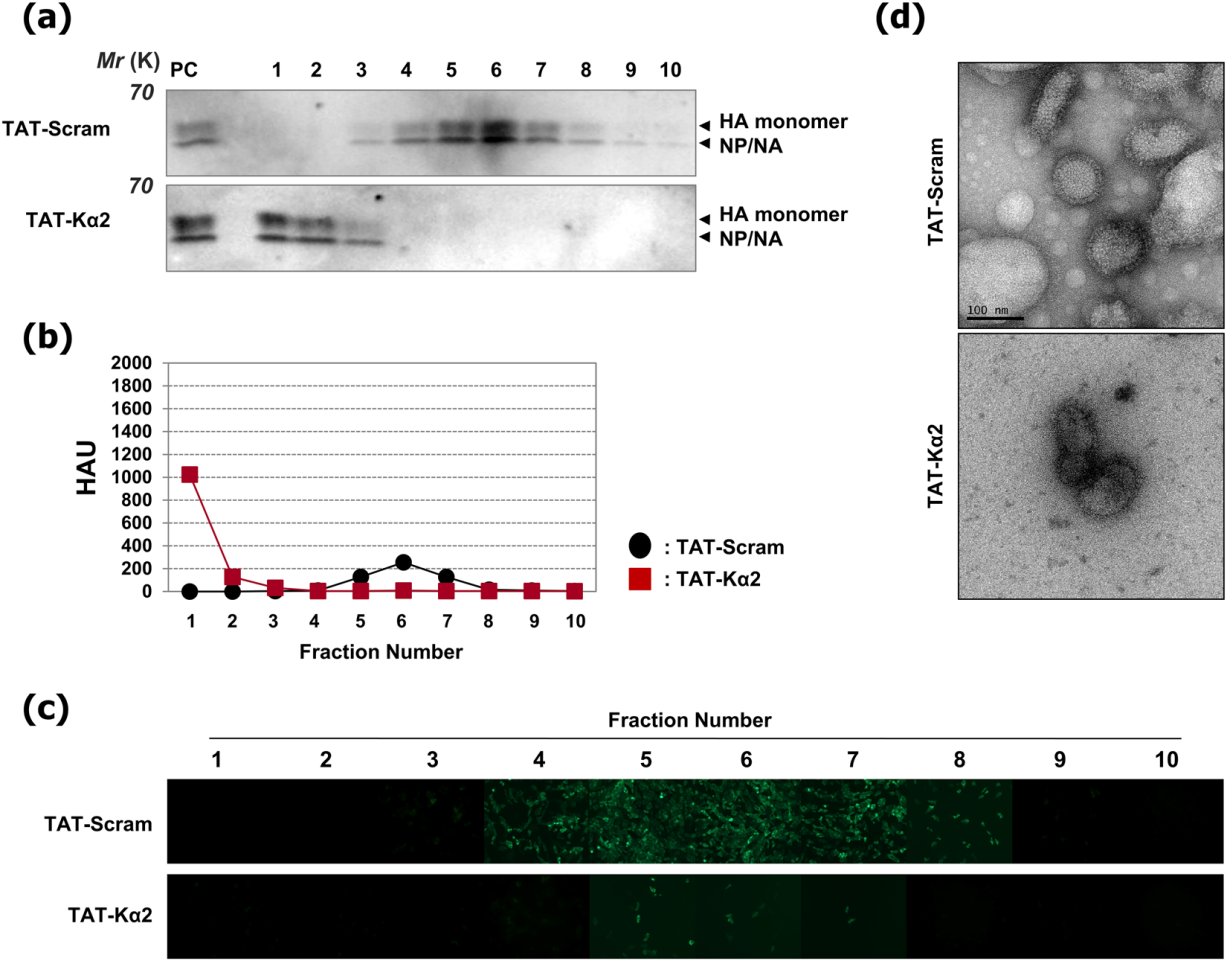
Velocity sedimentation ultracentrifugation. We investigated the biophysical properties of the PR8-GFP virus that had been treated with TAT-Kα2 peptide using the velocity sedimentation ultracentrifugation method to determine the virucidal mechanism of the TAT-Kα2 peptide. The virus was pre-incubated with the peptide (TAT-Kα2 or TAT-scramble) and loaded onto a continuous gradient, and fractions were collected as described above. (**a**) Immunoblotting of each fraction derived from a mouse that survived the PR8 challenge test with a polyclonal antibody. HA, NA, and NP proteins of the PR8 virus were detected. PC: wild-type PR8 virus was loaded. (**b**) Two-fold serially diluted fractions were tested for hemagglutination﻿. (**c**) MDCK cells were infected with each fraction to compare the infectivity of PR8-GFP viruses that were treated with each peptide. (**d**) Morphological differences in the virus particles between TAT-Kα2-treated virus and TAT-scramble peptide-treated virus were observed by TEM. Bar, 100 nm.

PR8-GFP virus was incubated with the TAT-Kα2 or the TAT-scramble for 20 min at RT; next, virus particles were analyzed by velocity sedimentation ultracentrifugation to provide information on the size and shape of the macromolecules in solution, and to identify how TAT-Kα2 disrupts or blocks influenza virus infection^19^. Fractions collected after ultracentrifugation were tested for viral components, hemagglutination and viral infectivity. Virus with no peptide treatment showed the same results as TAT-scramble-treated virus (data not shown). With TAT-scramble treatment, the viral proteins hemaglutinnin (HA), neuraminidase (NA), and nucleoprotein (NP) were detected in the centre fractions around the 40% sucrose cushion. However, with TAT-Kα2 treatment, these proteins were observed in the upper fractions around the 20% sucrose cushion (Figs 2a and S1B). Moreover, the hemagglutination assay yielded the similar results as immunoblotting (Fig. 2b). Although the TAT-Kα2 peptide-treated PR8-GFP virus contained 1,000 hemagglutinating units (HAU) in 100 μl of the upper fraction of the 20% sucrose cushion, the virus that was treated with the TAT-scramble peptide contained approximately 200 HAU in each positive fraction from the upper fraction of the 40% sucrose cushion to the upper fraction of the 50% sucrose cushion. Furthermore, the TAT-scramble-treated PR8-GFP viruses in the positive fractions around the 40% sucrose cushion showed high virus infectivity *in vitro*, but the TAT-Kα2 peptide-treated viruses in the upper fraction of the 20% sucrose cushion did not (Fig. 2c). With TAT-Kα2 treatment, a small number of the cells that were treated with the fraction corresponding to the 40% sucrose cushion showed fluorescence, possibly indicating infection by intact virus particles that had not interacted with TAT-Kα2. Therefore, we could hypothesize that the viruses obtained some infectivity defects through the contact with the TAT-Kα2 peptide. More importantly, the location of virus particles in the upper fraction of the 20% sucrose cushion indicated a loss of mass of the intact viruses through a destabilized or disrupted envelope structure. Taken together, the elicited results from the velocity sedimentation ultracentrifugation assay indicates that the TAT-Kα2 peptide might disrupt the envelope structure of the virus particles causing the virus to lose its infectivity, but the functional components remained intact.

For more evidence, PR8-GFP viruses that had been treated with the TAT-Kα2 peptide or the TAT-scramble peptide were observed by negative stain transmission electron microscopy (TEM). The virus treated with the TAT-Kα2 peptide did not have an intact envelope structure, whereas the TAT-scramble-treated PR8-GFP viruses showed a spontaneous lipid bilayer on their surfaces, similar to the normal influenza virus (Fig. 2d). Overall, all of the results indicated that the inhibition of virus infection was caused by the destabilization of the virus structure through the direct interaction with the TAT-Kα2 peptide.

### TAT-Kα2 peptide shows virucidal activity *in vivo*

**Figure 3:**
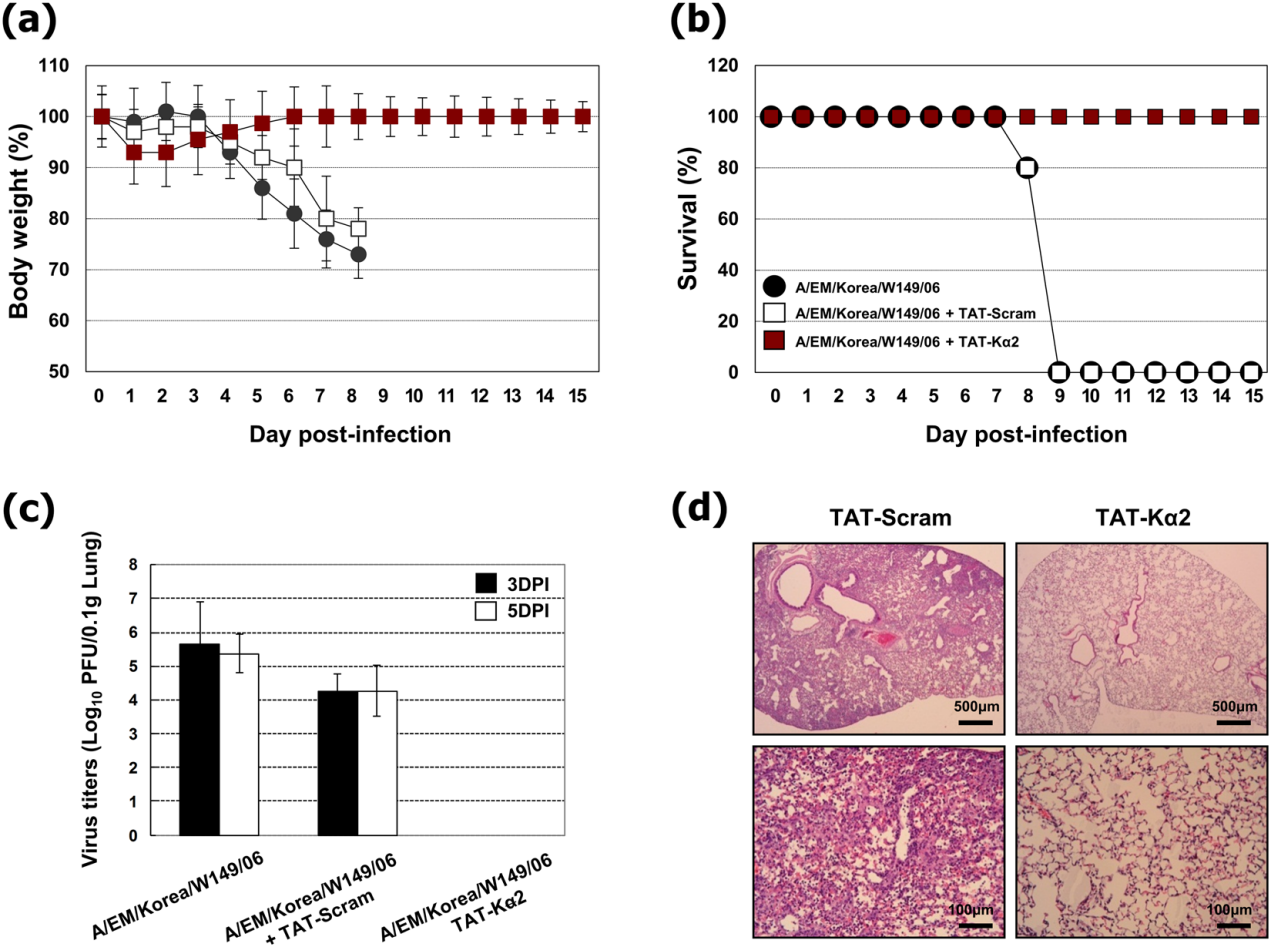
*In vivo* virucidal effect of the TAT-Kα2 peptide against HPAI (H5N1). The mice were inoculated with the W149 virus alone or mixtures of the W149 virus and each peptide (TAT-Kα2 or TAT-scramble peptides), and (**a**) percent body weight and (**b**) survival percentage were monitored for 15 days. (**c**) The lung virus titers of each group (three randomly selected mice from each group were sacrificed) are expressed as the means ± SD of the log_10_ plaque-forming units (PFU) per 0.1 g of tissue. The closed bar indicates the virus titer at 3 DPI, and the open bar indicates the virus titer at 5 DPI. (**d**) Histopathologic observation at 5 DPI. The upper panels were observed by light microscopy at 40× magnification, and the lower panels were observed at 200× magnification.

Mice were simultane﻿ously intranasally inoculated with 12 μM of TAT-Kα2 peptide and 10 MLD_50_ (50% of the mouse lethal dose) of the highly pathogenic avian influenza virus A/EM/Korea/W149/06 (W149, HPAI, H5N1) and were monitored for 15 days. Significantly different body weight percentages were observed among the groups from 5 days post-infection (DPI), and the differences were increased by more than 20% from 8 DPI (Fig. 3a). Moreover, the survival rate of the TAT-Kα2 peptide-treated mice was 100%, whereas none of the TAT-scramble peptide-treated mice survived on 9 DPI (Fig. 3b). At 3 and 5 DPI, three mice in each group were sacrificed for virus titration in the lungs. Mice that were treated with virus alone or virus with the TAT-scramble peptide contained very high titers of the W149 virus, and there was no significant difference between these groups. However, no virus was detected in the TAT-Kα2 peptide-treated group (Fig. 3c). Additionally, three mice in each group were sacrificed for histopathological observations of the lungs on 5 DPI. The TAT-Kα2 peptide-treated mice showed intact lung tissues with no virus in the lungs, whereas the alveoli of TAT-scramble peptide-treated mice were filled with a large number of inflammatory cells, including neutrophils and macrophages (Fig. 3d). Similar results in terms of body weight, survival and virus titre were obtained when the W149 virus was replaced with the PR8 (Fig. S2). According to the results of the W149 and PR8 experiments, we suggest that TAT-Kα2 peptide inhibits the virus activity and infectivity, and thereby prevents infection without any signs of toxicity in mice.

### The TAT-Kα2 peptide has virucidal activity against other enveloped viruses

**Figure 4:**
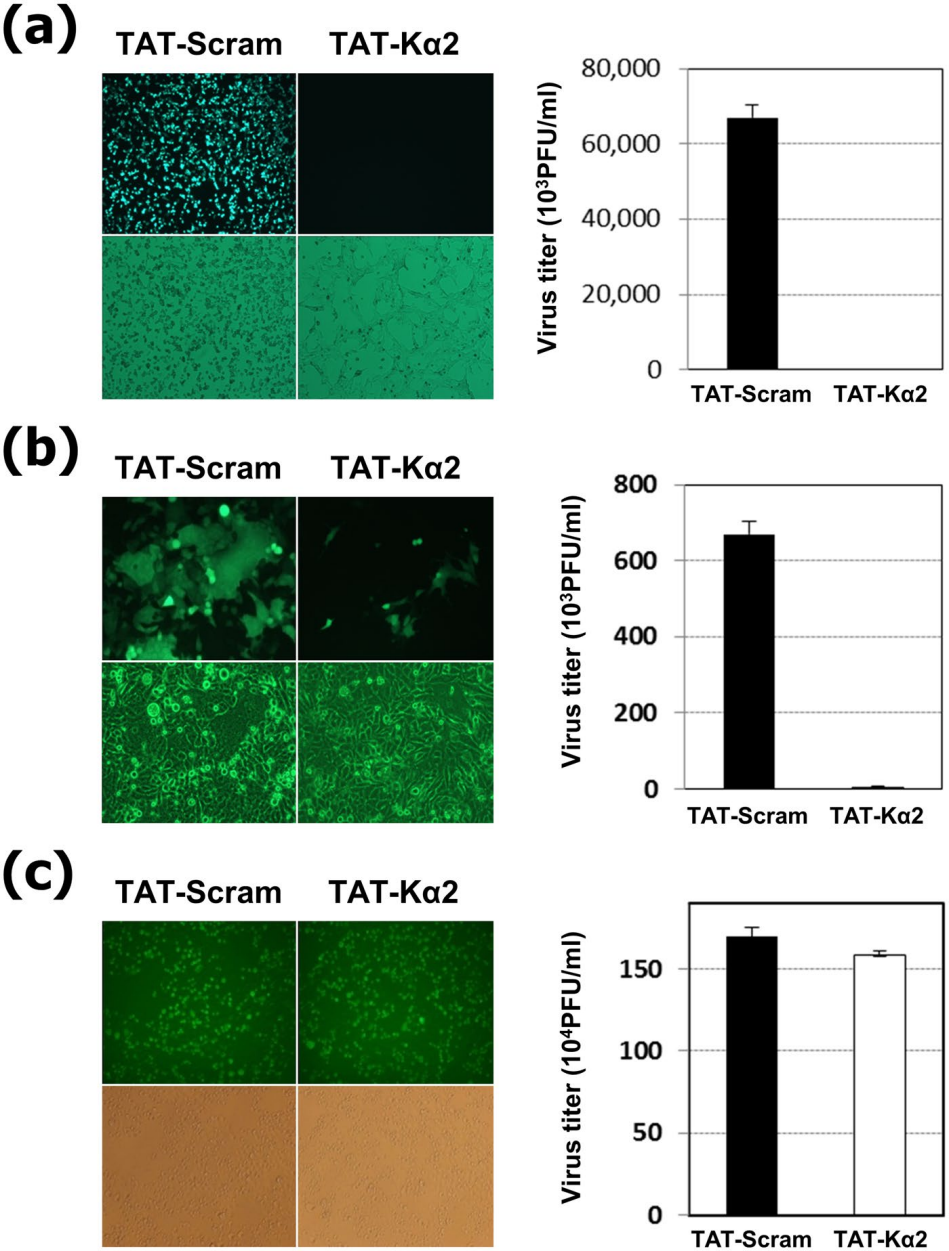
Broad-spectrum activity of the TAT-Kα2 peptide and its specificity. Enveloped viruses, (**a**) VSV (293T cells) and (**b**) RSV (Hep2 cells), and a non-enveloped virus, (**c**) Coxsackievirus (HeLa cells), were tested to determine the antiviral effects of TAT-Kα2 peptide against other viruses. The 40 μM of TAT-Kα2 peptide or TAT-scramble peptide was pre-incubated with the respective viruses, cell infection was determined by GFP expression, and the titers of each virus were determined.

The hypothesis that TAT-Kα2 exerted its antiviral activity by disruption of the viral envelope was further explored by testing its efficacy against other viruses (listed in Table 2). Infections with enveloped viruses, such as vesicular stomatitis virus (VSV) (Fig. 4a) and respiratory syncytial virus (RSV) (Fig. 4b), were inhibited by the TAT-Kα2 peptide, whereas the TAT-scramble peptide could not inhibit infections with these viruses. However, the TAT-Kα2 peptide did not show any virucidal effects against non-enveloped viruses, such as Coxsackievirus and Enterovirus 71, similar to the TAT-scramble peptide (Table 2 and Fig. 4c). Furthermore, the half maximal inhibitory concentration (IC_50_) of the TAT-Kα2 peptide against enveloped viruses indicated that the peptide had significant virucidal effects at very low concentrations: 0.5–1.2 μM for influenza viruses and 0.8–5.7 μM for other enveloped viruses (Table 2). Taken together, these data suggest that enveloped viruses were inhibited by the TAT-Kα2 peptide, and significant antiviral activity of this peptide was observed, even at low inhibitory concentrations. These results and the mechanistic study indicate that the TAT-Kα2 peptide can suppress the infection of various enveloped viruses through a direct interaction with the viral envelope and acts as a destabilizer of virus particles. Notably, TAT-Kα2 provides broad cross-protection against diverse subtypes of influenza viruses in both groups 1 and 2, as shown in Table 2.

**Table 2:** Virocidal effect of TAT-Kα2 peptide against various viruses.

Virus	Enveloped	Genome	IC_50_(μM)
Vesicular stomatitis virus	Envelope	RNA(−)	2.9
Newcastle disease virus	Envelope	RNA(−)	0.8
Respiratory Syncytial Virus	Envelope	RNA(−)	3.4
Parainfluenza virus	Envelope	RNA(−)	2.4
Influenza virus (H1N1) A/Puerto Rico/8/34	Envelope	RNA(−)	0.5
Influenza virus (H5N2) A/bird/Korea/W81/2005	Envelope	RNA(−)	0.8
Influenza virus (H7N3) A/Aquatic bird/Korea/W44/2005	Envelope	RNA(−)	1.2
Influenza virus (H9N2) A/Chicken/Korea/116/2004	Envelope	RNA(−)	0.7
Porcine epidemic diarrhea virus	Envelope	RNA(+)	3.5
Herpes simplex virus	Envelope	DNA	5.7
Coxsakievirus	Naked	RNA(+)	No
Enterovirus71	Naked	RNA(+)	No

### Specific amino acid residues are critical for the virucidal effect of the TAT-Kα2 peptide

**Figure 5:**
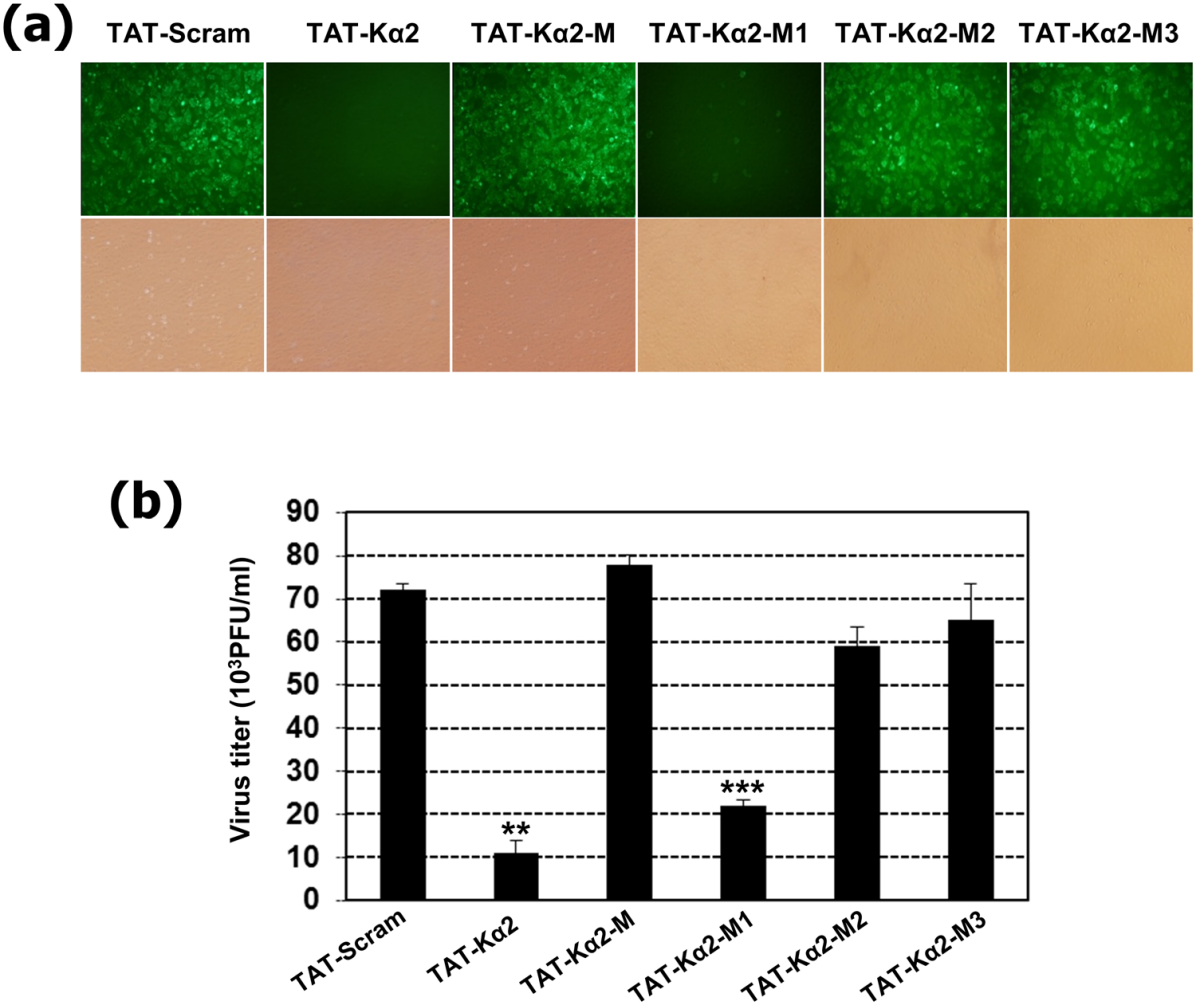
Screening for critical amino acid (aa) residues in the TAT-Kα2 peptide that are responsible for its virucidal effect. As listed in Table 1, TAT-scramble, TAT-Kα2, and four different alanine mutant (M, M1, M2 and M3) peptides were compared to identify the critical aa residues that were responsible for the antiviral ability. (**a**) Infectivity of the PR8-GFP virus after treatment with each peptide (40 μM). The upper panels show the fluorescence microscopy observations and the lower panels show the light microscopy observations at 200× magnification. (**b**) Virus titers after treatment with each peptide. Virus titers are expressed as the means ± SD of 10^3^ PFU/ml.

Four peptides varients of TAT-Kα2, M, M1, M2 and M3, were synthesized (Table 1) to identify the critical amino acids for inhibition of viral infection. All three of the hydrophobic core residues, Leu17, Phe18, and Leu19, were replaced with alanine (Ala) and denoted as M1, M2, M3 and M, respectively. The antiviral effects of all four alanine-mutated peptides were compared with the TAT-Kα2 and TAT-scramble peptides. As shown in Fig. 5a, the TAT-Kα2 and TAT-Kα2-M1 peptides significantly inhibited PR8-GFP virus infection compared to other alanine substitution mutants. The numeric values of these results are also shown using a plaque assay (Fig. 5b). When the amino acids Phe18 (M2) or Leu19 (M3) were altered, the antiviral efficacy of the peptide was decreased by three-fold, suggesting that these two amino acid residues are critical for the antiviral activity of the TAT-Kα2 peptide. Although Leu17 seemed less important, the presence of this amino acid residue induced a two-fold increase in the virucidal effect. On the basis of these observations, Phe18 and Leu19 in the Kα2 portion of the peptide seem to have the most important roles in disruption of the the viral envelope, and in turn, inhibition of virus infection.

### The TAT-Kα2 peptide is safe and effective under various conditions

The cytotoxicity of the TAT-Kα2 peptide was tested both *in vitro* and *in vivo* to determine the safety of different concentrations of the peptide. No cytotoxicity or CPE was observed with the concentration of 40 µM of the TAT-Kα2 peptide used (Fig. 1d). Additionally, no signs of toxicity were observed in the TAT-Kα2 peptide-treated mice during the *in vivo* challenge test (data not shown). Furthermore, the peptide was tested *in vitro* under different conditions, including various pre-incubation times of peptide and PR8-GFP virus prior to cell treatment, different pre-incubation temperatures, and different peptide doses to determine the most efficient virucidal activity of the TAT-Kα2 peptide. First, the minimal pre-incubation time needed to elicit significant antiviral activity of the TAT-Kα2 against IAV was determined. As shown in Fig. S3, the infectivity of the PR8-GFP virus was dramatically suppressed by incubation with the TAT-Kα2 peptide within 5 min, and no viral infection was detected from 15 min onwards. This result suggests that TAT-Kα2 requires only a very short period of contact with the virus to inactivate infectivity. Second, the test was performed at 4, 22 and 37 °C to determine the effective activation temperature of the TAT-Kα2 peptide. RT (22 °C) and the human body temperature (37 °C) showed efficient virucidal effects of the TAT-Kα2 peptide, whereas low pre-incubation temperature (4 °C) did not show significant activity of the peptide (Fig. S4). As shown in Fig. S5, dose-dependent virus inhibition was observed, with an approximately 40% reduction in the virus titer at 0.5 μM TAT-Kα2, 80% inhibition at 1 μM TAT-Kα2, 95% inhibition at 2 μM TAT-Kα2, and almost complete inhibition at 8 μM TAT-Kα2 compared with the TAT-scramble control. These results demonstrated that TAT-Kα2 peptide can be safely used in the range of 0.5–8 μM and can elicit significant antiviral activity within 5 min at RT and body temperature.

## Discussion

Vaccination is still the most promising treatment for controlling viral diseases^20^, however, currently only a few vaccines are available to control pandemic influenza virus infections in humans^21–23^. Therefore, alternative methods, including therapeutic medicine, passive monoclonal antibody therapy and gene therapy, are also desired^24–26^. Previously, the regulatory function of the FLIP-derived peptide Kα2 was described in the context of induction of autophagy for cancer therapy^18^. In addition to the robust role of the TAT-Kα2 peptide in autophagy induction, here we evaluated its virucidal activity on the basis that several α-helical peptides are known to display virucidal activity, disrupting viral envelopes^3, 27^.

Because, one of the main obstacles in the clinical use of therapeutic peptides is cytotoxicity, we rigorously investigated the potential cytotoxicity of the TAT-Kα2 peptide. Here, the TAT-Kα2 peptide showed no cytotoxicity or CPE at concentrations up to 100 μM, and the peptide elicited a significant anti-influenza effect at very low dose, producing over 95% of inhibition at 2 μM (Fig. S5). Moreover, no signs of toxicity were observed in mice in the challenge test. Therefore, these results suggest that the TAT-Kα2 peptide can be safely used with maximum efficacy, subject to confirmation by detailed *in vivo* studies.

TAT-Kα2 showed cross-group protection for both group 1 (H1, H5 and H9) and group 2 (H7) IAVs (Table 2), and also inhibited infection by other enveloped viruses. However, TAT-Kα2 peptide is different from other anti-influenza peptides, such as entry blocker (EB) and killer decapeptide (KP), because TAT-Kα2 does not seem to have any specific interactions with influenza components, such as HA and Matrix 1 proteins^28, 29^. The antiviral specificity of the TAT-Kα2 peptide is more likely similar to that of the C5A peptide, which has an α–helical structure, and which recognises lipid components of enveloped viruses to destabilise their liposome membranes^30, 31^. This recognition may be the reason why the TAT-Kα2 peptide also inhibits infection by other enveloped viruses. In contrast to the results with the C5A peptide^30^, we observed robust inhibition of various IAV subtypes at very low doses of TAT-Kα2. These comparisons suggest that the TAT-Kα2 has great promise as a means to control diseases caused by influenza virus and other enveloped viruses, without problems relating to safety or resistant isolates.

In support of this notion, we performed a velocity sedimentation ultracentrifugation analysis. In this structural activity analysis, we observed that TAT-Kα2 peptide directly interacts with the viral envelope and destabilizes the viral structure. Additionally, in hemagglutination assays with the sedimentary virus particles from each fraction, HA proteins displayed on the surface of the viral envelope retained their own function to agglutinate red blood cells (RBCs). This result supports our hypothesis that the peptide seems to interact with the viral liposome membrane instead of envelope proteins.

The TAT portion of the TAT-Kα2 peptide, which was derived from the HIV-1 TAT protein transduction domain, functions to deliver the TAT-fused peptide (TAT-Kα2) into liposome membranes^18, 32–35^, however, TAT alone does not have any antiviral activity^28^, which was confirmed by our results. The antiviral component of the TAT-Kα2 peptide is the Kα2 portion. The membrane targeting property of TAT and the α-helical structure of the Kα2 peptide [Protein Data Bank (PDB) ID code 3CL3]^36^ suggests that TAT-Kα2 should presumably interact with the viral envelope, leading to membrane permeation by transmembrane pore formation via a barrel-stave mechanism or membrane destruction via a carpet-like mechniasm^27^. The precise mechanism should be elucidated in future studies.

In negative -stain TEM observations of peptide-treated IAV, we found that virus particles that were disrupted by the TAT-Kα2 had a collapsed lipid-bilayer membrane structure, indicating that the destabilizing activity of TAT-Kα2 peptide might be the main cause for the loss of viral infectivity. Additionally, as reported in the previous study by Lee *et al*.^18^, the hydrophobic core residues of Kα2 (Leu-Phe-Leu) played a critical role in its function, which was again revealed in this study through the alanine mutation assay. Although it is still important to investigate how these residues interact with the viral envelope through a crystal structure analysis, our alanine mutation assay results suggest that the hydrophobic core residues Phe18 and Leu19 have critical roles in destabilizing the viral envelope structure.

In addition to the previously reported autophagy activity^18^, we have now identified another role of the TAT-Kα2 peptide: the inhibition of enveloped virus infections both *in vitro* and *in vivo*. It is not yet clear whether the autophagy and the virucidal functions of Kα2 both result from the same functional domain of the peptide, or whether they are both mediated by membrane association. Here, we specifically confirmed that TAT-Kα2 inhibits infectivity of various subtypes of influenza viruses across groups 1 and 2, along with several other enveloped viruses. Our results have demonstrated that the antiviral mechanism of TAT-Kα2 involves direct interaction with the viral envelope as a membrane-targeting agent. We have also shown that, to inhibit infectivity, the peptide has to be incubated with the virus prior to or at the same time as attachment to the cell. Though the peptide has to be added simultaneously to disrupt virus membrane will limit some of the peptide applications, TAT-Kα2 can considered to be a promising antiviral agent candidate for controlling or inhibiting various subtypes of the influenza virus and other disease-causing enveloped viruses.

## Meterials and Methods

### Viruses and cells

A/PR/8/34 and green fluorescent protein (GFP) tagged A/PR/8/34 were provided by Dr. Jae U. Jung of University of Southern California to conduct this study. The highly pathogenic avian influenza virus, A/EM/Korea/W149/06 was isolated from fecal specimens of Korean wild bird habitats^37^, and used for the challenge test. To check the virocidal effect of TAT-Kα2 peptide against different viruses, Vesicular stomatitis virus, Newcastle disease virus, Respiratory syncytial virus, Parainfluenza virus, Porcine epidemic diarrhea virus, Coxsakievirus, Enterovirus 71, A/bird/Korea/W81/2005 (H5N2), A/Aquaticbird/Korea/W44/2005 (H7N3), and A/Chicken/Korea/116/2004 (H9N2) were used in this study. MDCK cells were grown in minimum essential medium (MEM) with Eagle salts containing 10% fetal bovine serum and incubated at 37 °C in 5% CO_2_. Those cells were used to confirm GFP tagged virus replications and plaque assay for virus titration.

### Peptides

All peptides tested in this study were commercially synthesized by Peptron Cooperation (Republic of Korea), and were highly purified (over 95% purity grade). These synthesized peptides were reconstituted in 100% dimethylsulfoxide (DMSO), then dissolved in 0.5% DMSO as a working stock and stored at −80 °C until used.

### Determination of inhibition concentration 50% (IC_50_)

IC_50_ values of TAT-Kα2 peptide against various enveloped and non–enveloped viruses were determined as follows. Each dose of TAT-Kα2 peptide (0.5, 1, 2, 4, and 8 μM) were pre-incubated with 0.5 MOI of viruses listed in Table 1 for 20 min at RT. The pre-incubated mixtures were added to each susceptible cell for each virus. After 2 h, the wells were washed using phosphate buffered saline (PBS). Then the cell growth media (containing 10% FBS) were added and incubated for 48 h at 37 °C with 5% CO_2_. CPE of the each well was observed to determine the IC_50_ value using Reed and Muench method^38^.

### *In vitro* cytotoxicity assay

The cytotoxic effect of MDCK cell monolayers were determined by quantifying the cell viability using trypan blue exclusion test as described previously^39^. Different concentrations (5, 10, 20, 40, 80, 100 µM) of TAT-Kα2 was added to the each well after cells grown in 72-well plates into a 75–80% confluent monolayer, and all the plates were incubated at 37 °C in a humidified 5% CO_2_. As a control, the dilution medium without the samples was used. Clarified cells were then stained with 0.4% trypan blue (Invitrogen) (ratio: 1:1) and percentages of viable cells were counted after mounted to the hemocytometer.

### Animals and HPAI virus challenge

All animal experiments were managed in strict accordance with the Guide for the Care and Use of Laboratory Animals from the US National Institutes of Health and performed in BSL-3 laboratory facilities with the approval of the Institutional Animal Care and Use Committee of Bioleaders Corporation (Reference number BLS-ABSL-10-003).

Before the test, the 5﻿-week-old female BALB/c mice were confirmed as seronegative for influenza A virus by hemagglutination inhibition (HI) assay using HPAI H5N1 (A/EM/Korea/W149/06) or H1N1 PR8 (A/PR/8/34) virus. Three experimental groups of 19 heads each were prepared and intranasally inoculated with 30 μl of each inoculum: 0.85% saline + virus; 10 MLD_50_, TAT-scramble peptide + virus; 10 MLD_50_, or TAT-Kα2 peptide + virus; 10 MLD_50_. The amount of H5N1 and H1N1 PR8 mixed with the Kα﻿2 peptide was 8.24 × 10^1^ and 1.04 × 10^4^ PFU/mouse, respectively. After inoculation to the mice, body weight and survival percentage of mice were monitored for 15 days. Three heads from each group were randomly sacrificed to determine the lung virus titer at 3 and 5 DPI and histopathology of the lungs at 5 DPI. The remaining 10 heads from each group were used to check survival rate.

### Titration of virus in lung

The lungs of mice at 3 and 5 DPI were taken and homogenized in 500 µl PBS containing the following antibiotics: 200 U penicillin (Sigma-Aldrich, USA), 200 µg streptomycin sulfate (720 U/mg, Sigma-Aldrich, USA) and 68 µg amphotericin B (approximately 80% by HPLC, Sigma-Aldrich, USA). Tissue homogenates were centrifuged at 10,000 × g for 10 min and supernatants were transferred to new sterile micro centrifuge tubes. This clarification was repeated twice for the plaque assay. The prepared samples for the plaque assay were serially diluted 10-fold in serum-free GIBCO^TM^ MEM (Invitrogen, USA) and inoculated on MDCK cells that were grown to confluence in 6 well TC plates. After 1 h incubation at 37 °C with 5% CO_2_, inoculums were discarded and serum-free GIBCO^TM^ minimum essential alpha medium (Invitrogen, USA) containing 0.9% agar (Sigma-Aldrich, USA), 0.01% neutral red solution (Merck, Germany), and 1 µg/ml of L-1-tosylamide-2-phenylmethyl chloromethyl ketone (TPCK) treated trypsin (Invitrogen, USA) was over-laid. Upon the appearance of virus plaques at 48 h post infection, the wells were fixed with 5% formaldehyde (Merck, Germany) and stained with crystal violet (Sigma–Aldrich, USA). The plaques were counted and the virus titers in the lung were expressed as log_10_ PFU per 0.1 g of tissue collected and analyzed according to the report of Charles *et al*., with some modifications^40^.

### Histopathological observations

The extracted lungs at 5 DPI from mice challenged with A/EM/Korea/W149/06 virus were fixed in 10% neutral-buffered formalin for histopathological observations. They were embedded in paraffin, sectioned (4 µm), stained using standard hematoxylin and eosin staining methods, and examined under light microscopy (magnification, ×200).

### Virus observation by negative stain transmission electron microscopy

The clarified allantoic fluids containing propagated PR8 virus from 10 day-old specific pathogen-free (SPF) eggs were loaded on 20% sucrose cushion and purified by ultracentrifugation at 82,592 × g for 2 h in a SW-28 rotor (Beckman, USA). After virus titration by hemagglutination assay, 10 μl of PR8 virus (2 HAU/ml) was mixed with 20 μl of PBS or 20 μl of PBS containing TAT-Kα2 peptide (1 μg/μl). Immediately, 10 μl in each of these two specimens were taken and mixed with 10 μl of 1% uranyl acetate negative staining solution. Afterward, a drop of each specimen was loaded onto a Formvar- or carbon-coated Formvar hexagonal 300 mesh copper grids and air-dried for a few minutes. Two prepared specimens were observed under Tecnai^TM^ G^2^ Spirit bio-Twin transmission electron microscope (FEI Company, USA) by an accelerating voltage of 80 kV.

### Sedimentation of TAT-Kα2 peptide treated virus

The concentrated GFP-tagged PR8 virus (≥14 log_2_ HAU/ml) was exposed to the peptide by mixing and loaded over a 20–60% sucrose gradient. After ultracentrifugation at 82,592 × g for 2 h in a SW-28 rotor, 3 ml of upper layers and 3 ml of the lower layers in each of different sucrose percentage were collected (total 10 fractions) and examined which fraction contains the viral components. The GFP-tagged PR8 viruses in each fraction were detected by various methods including immunoblot using anti-PR8 virus polyclonal mouse antibody derived from PR8 virus infected mouse, SDS-PAGE followed by silver staining, hemagglutination assay and through the observation of GFP expression after infection to MDCK cells.

### Statistical analysis

All *in vitro* assays were carried out in triplicate and the data were expressed means ± standard deviations (SD). Differences were examined using student t-test and p-value < 0.05 was regarded as significant.

## Electronic supplementary material

Supplementary info
