# Supplementary material for: Inhibition of highly pathogenic avian influenza (HPAI) virus by a peptide derived from vFLIP through its direct destabilization of viruses
Source: Sci Rep. 2017 Jul 7;7:4875. doi: 10.1038/s41598-017-04777-4 (PMC5501782; doi:10.1038/s41598-017-04777-4)
Supplement: Supplementary file 1 — Supplementary info [file 41598_2017_4777_MOESM1_ESM.pdf]

Inhibition of highly pathogenic avian influenza (HPAI) virus by a peptide derived from vFLIP through its direct destabilization of viruses

Ho-Jin Moon, Chamilani Nikapitiya, Hyun-Cheol Lee, Min-Eun Park, Jae-Hoon Kim, Tae-Hwan Kim, Ji-Eun Yoon, Won-Kyung Cho, Jin Yeul Ma, Chul-Joong Kim, Jae U. Jung, and Jong-Soo Lee

Supplementary information

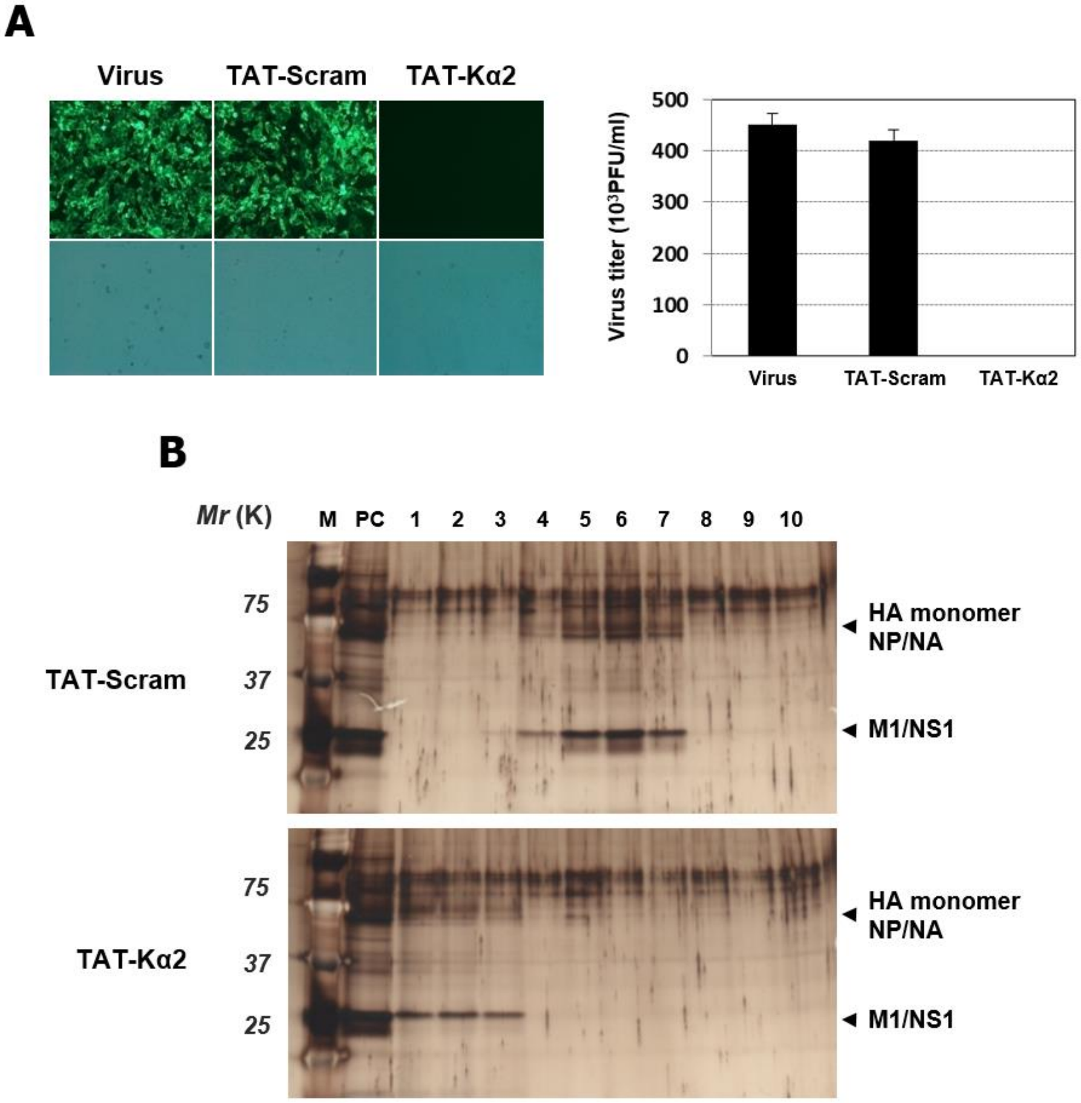

Figure S1. TAT-Kα2 inhibits influenza virus infection by directly interacting with the virus particle. (A) As a pilot study, the TAT-Kα2 peptide or TAT-scramble peptide was used to treat the PR8-GFP virus to observe the virucidal effect of Kα2 (12 μM). The upper panels show the observations of GFP expression, and the lower panels show the observations with normal light microscopy and a blue filter. The virus titers are expressed as the means ± SD of three independent assays. (B) Silver staining of each fraction derived from velocity sedimentation ultracentrifugation. In addition to the immunoblotting results shown in Fig. 2A, we repeated this experiment to confirm the result using the silver staining method. HA, Hemagglutinin; NP, Nucleoprotein; NA, Neuraminidase; M1, Matrix protein 1; NS1, Nonstructural protein 1.

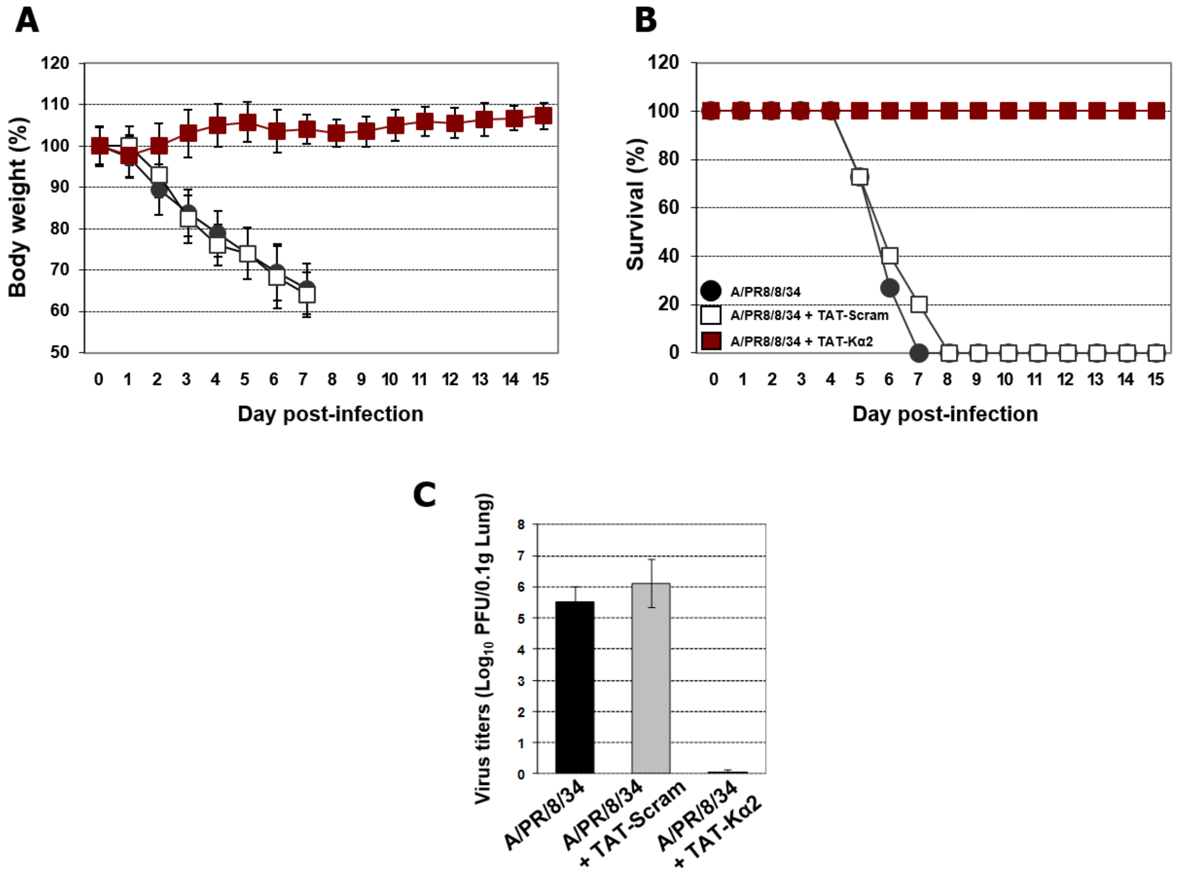

Figure S2. *In vivo* virucidal effect of the TAT-Kα2 peptide on the PR8 virus (H1N1). Mice were challenged with PR8 virus in the same manner as the W149 challenge test to confirm the broad-spectrum activity of the TAT-Kα2 peptide *in vivo*. (A) Body weight. After 7 DPI, the percent body weights of the virus only and TAT-scramble groups are not displayed owing to the mortality of these groups. (B) Percent survival. (C) Virus lung titers at 5 DPI. In each group, three randomly selected mice were sacrificed to extract lungs.

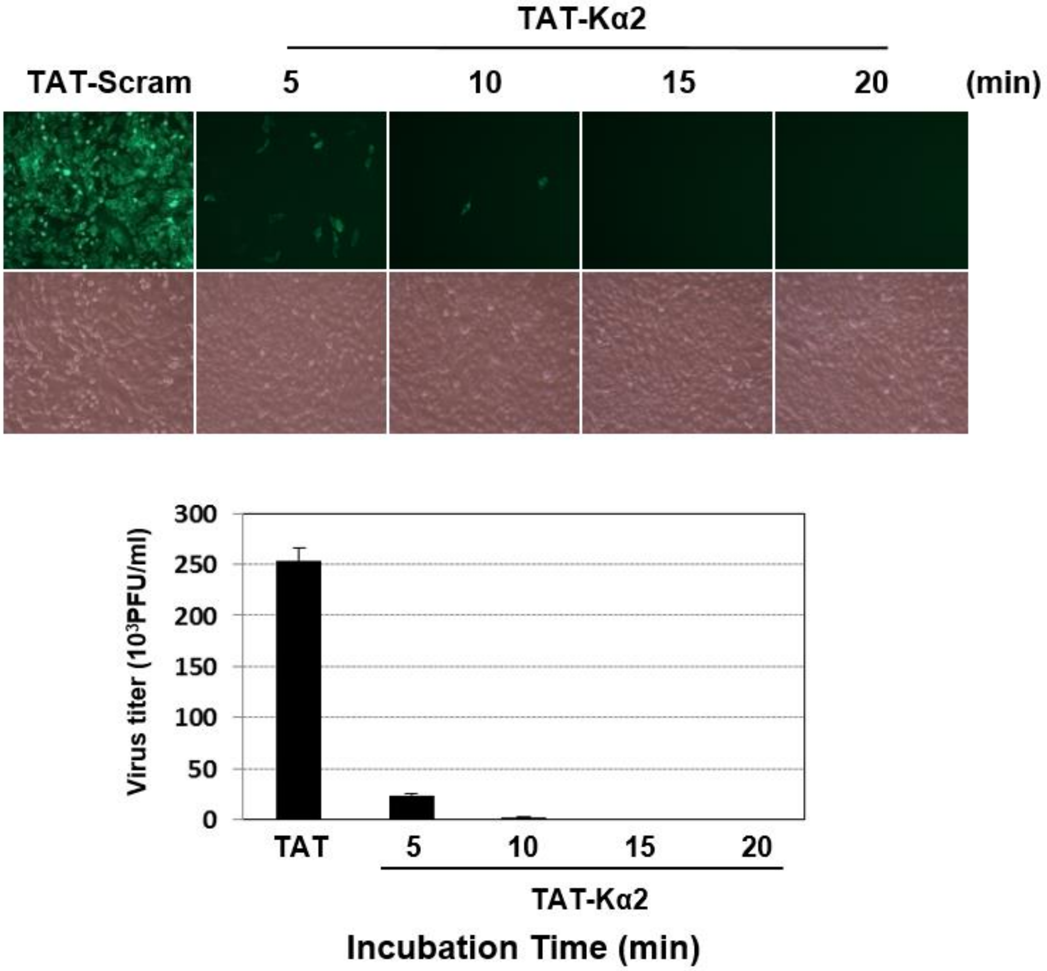

Figure S3. Antiviral effect of the TAT-Kα2 peptide on the PR8-GFP virus after various preincubation periods. The peptide was pre-incubated with 12 μM of the PR8 virus for 5, 10, 15, or 20 min, and the effects were compared to determine the optimal pre-incubation time for the virucidal effect of TAT-Kα2. The TAT-scramble peptide did not show antiviral effects at all pre-incubation times; thus, only a single representative titer was displayed in the graph. The infected cells and viral GFP expression were observed by microscopy. Virus titers are expressed as the means ± SD of 10^3 PFU/ml.

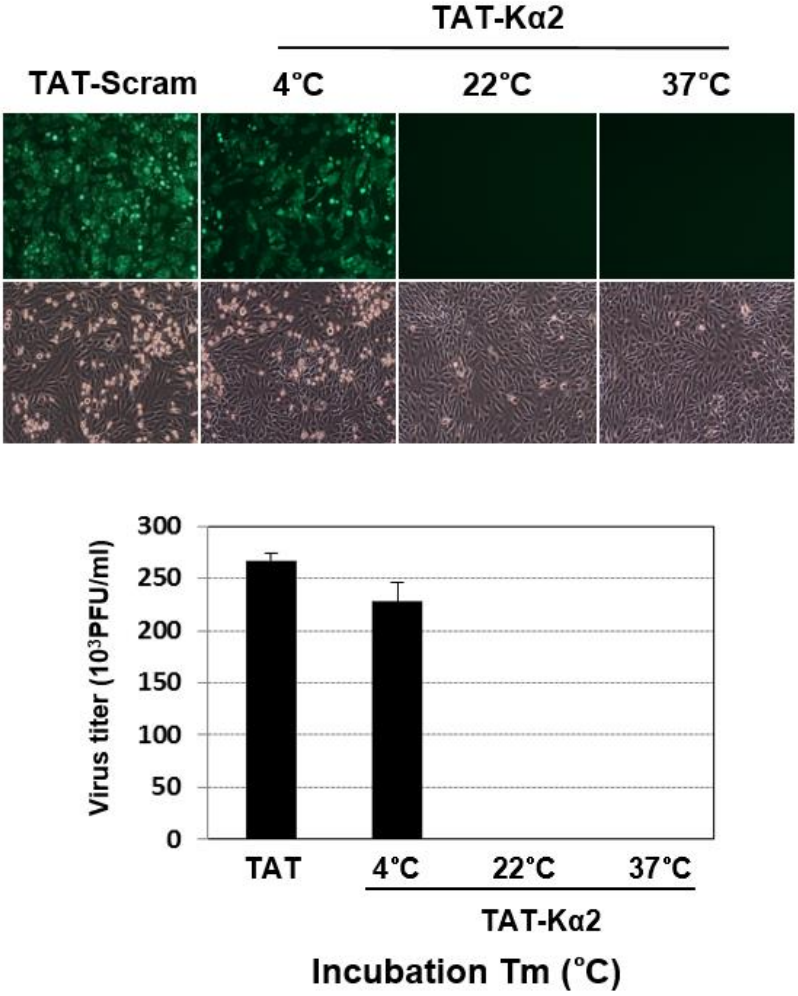

Figure S4. Antiviral effect of the TAT-Kα2 peptide on the PR8-GFP virus at various pre-incubation temperatures. The PR8-GFP virus and peptides were pre-incubated at various pre-incubation temperatures, 4°C, 22°C (representative room temperature), and 37°C (representative human or host animal body temperature), and the virus infectivity was observed by microscopy to determine the optimal temperature for the antiviral effects of TAT-Kα2. Additionally, only the average titer of the GFP expression of the TAT-scramble peptide-treated PR8 virus is shown in the figure owing to its consistency under various conditions. The viral titers are expressed as the means ± SD of 10^3 PFU/ml.

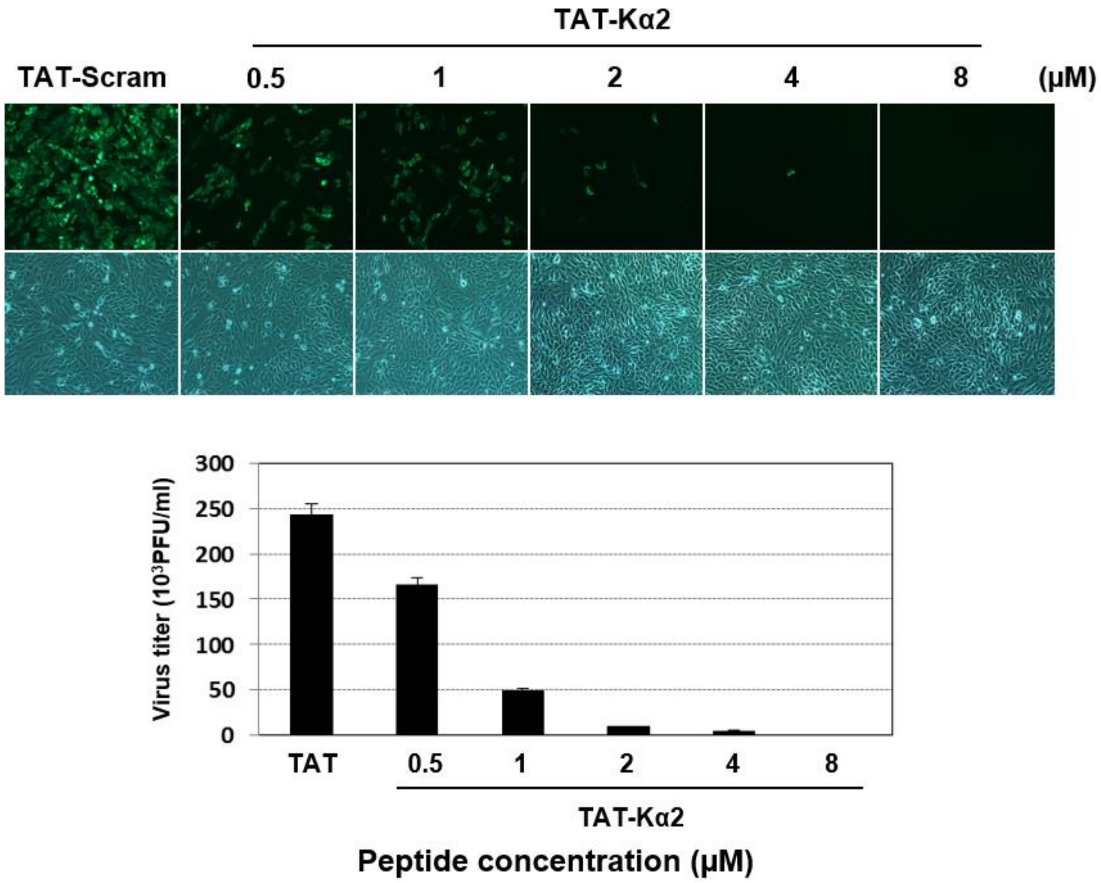

Figure S5. Determination of the optimal dose of the TAT-Kα2 peptide required for its virucidal effect. Various amounts (0.5, 1, 2, 4, and 8 μM) of the TAT-Kα2 and TAT-scramble peptides were tested against the GFP-tagged PR8 virus to determine the optimal dose required for a significant antiviral effect. There were no major differences at any of the TAT-scramble peptide doses; hence, only one representative condition is shown. The infected cells and viral GFP expression were observed by microscopy. The virus titers are expressed as the means ± SD of 10^3 PFU/ml.

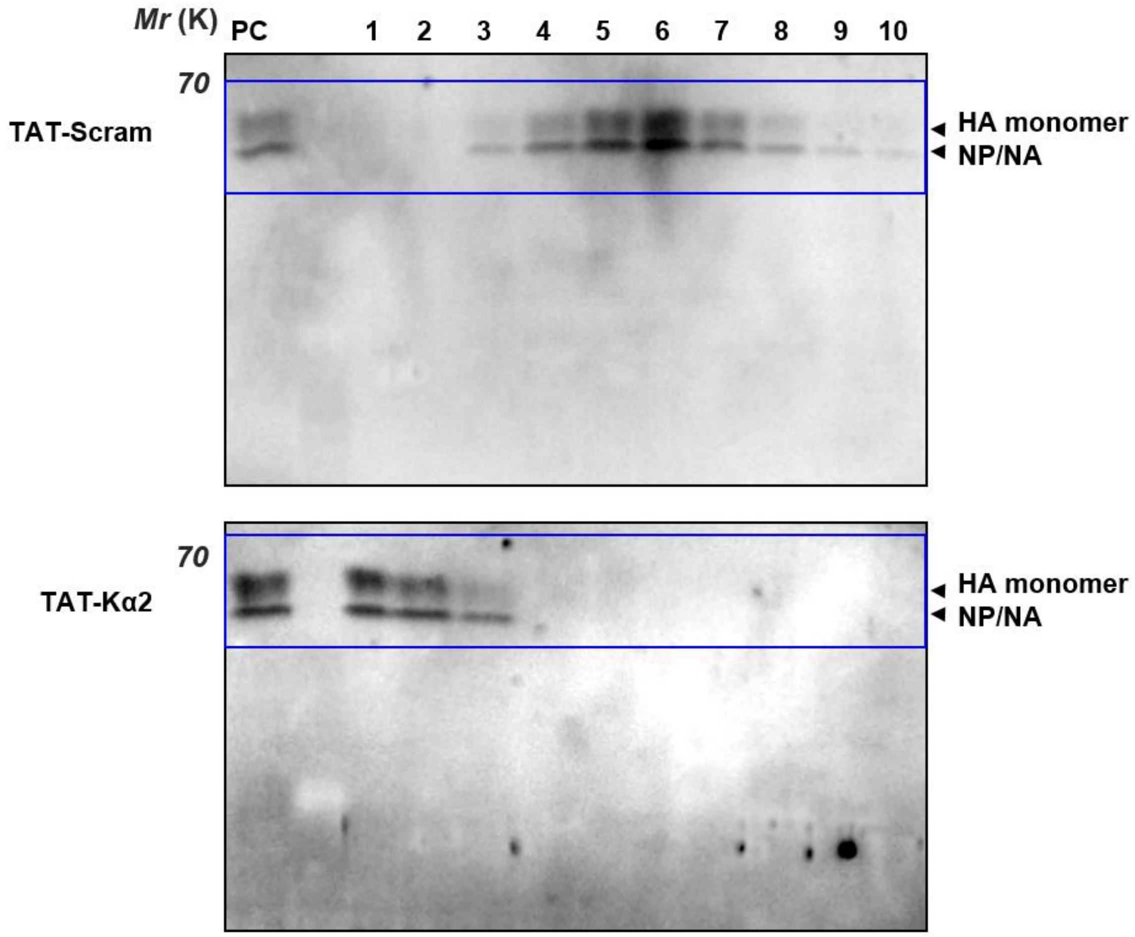

Figure S6. Full length blot cropped for Figure 2A
